# Performance of Orthopaedic Shoulder and Elbow Surgeons on a Biostatistical Knowledge Examination

**DOI:** 10.1155/2023/8840263

**Published:** 2023-09-11

**Authors:** Andrew P. Collins, Max McCall, Joshua Cassinat, Alison Grise, Jonathan Schwartzman, John Kelly, Benjamin C. Service

**Affiliations:** ^1^University of Washington Orthopaedics and Sports Medicine, Seattle, Washington 98195, USA; ^2^Emory University School of Medicine Department of Orthopaedics, Atlanta, Georgia 30329, USA; ^3^University of Central Florida College of Medicine, Orlando, Florida 32827, USA; ^4^Orlando Health Jewett Orthopedic Institute, Orlando, Florida 32806, USA

## Abstract

**Background:**

The objective of this study is to evaluate the biostatistical interpretation abilities of fellowship trained orthopaedic surgeons.

**Methods:**

A cross-sectional survey was administered to orthopaedic surgeon members of the American Shoulder and Elbow Surgeons (ASES), assessing orthopaedic surgeon attitudes towards biostatistics, confidence in understanding biostatistics, and ability to interpret biostatistical measures on a multiple-choice test.

**Results:**

A 4.5% response rate was achieved with 55 complete survey responses. The mean percent correct was 55.2%. Higher knowledge test scores were associated with younger age and fewer years since board exam completion (*p* ≤ 0.001). Greater average number of publications per year correlated with superior statistical interpretation (*p*=0.009). Respondents with higher self-reported confidence were more likely to accurately interpret results (*p* ≤ 0.017). Of the respondents, 93% reported frequently using statistics to form medical opinions, 98% answered that statistical competency is important in the practice of orthopaedic surgery, and 80% were eager to continue learning biostatistics.

**Conclusions:**

It is concerning that fellowship-trained shoulder and elbow surgeons, many of whom frequently publish or are reviewing scientific literature for publication, are scoring 55.2% correctly on average on this biostatistical knowledge examination. Surgeons that are further from formal statistical knowledge training are more likely to have lower biostatistical knowledge test scores. Respondents who published at the highest rate were associated with higher scores. Continuing medical education in biostatistics may be beneficial for maintaining statistical knowledge utilised in the current literature.

## 1. Introduction

Evidence-based medicine (EBM) relies on physicians to have a deep understanding of the literature. Although some clinical practice guidelines relay bottom-line summaries of relevant research [1, 2], many clinical questions must be answered by accessing original research [3]. This process calls for physicians to critically evaluate research quality, including study design, conduct, and analysis. More importantly, and perhaps most challenging, physicians must determine how the research applies to their own practice.

While distinct from orthopaedic surgeons and our study population, reports have shown that practicing physicians, especially those who lack formal training in biostatistics and epidemiology, had an overall poor understanding of routine statistical terms and a limited ability to interpret study results [4–6]. The majority of medical schools have since incorporated basic biostatistics courses into their curriculum [7]; however, over that same time, an increased focus on academia has led to a surge in publications [8, 9]. As a result, authors have integrated complex statistical methods in an effort to set themselves apart [10]. These issues increase the difficulty for reviewers to appraise research methodology in studies, and it is plausible that researchers intentionally complicate their methodology to push past reviewers. In a letter to the editor, Horton and Switzer [10] reported that statistical methods used in published works between 2004 and 2005 increased in complexity. Specifically, of the methods they observed, only 21% would be expected to be covered in an introductory statistics course.

As part of the American Board of Orthopaedic Surgery (ABOS) Part I accrediting examination, orthopaedic surgeons may encounter 0.5–1.5% of questions referencing biostatistics [11]. The ABOS exam tests the interpretation of epidemiologic information, associations, health impact, study design and interpretation, types of observational studies, sampling and sample size, subject selection, exposure allocation, hypothesis testing, and statistical interference [11]. Additionally, recertification examinations place an important focus on current literature and guidelines [12]. Despite the decisions physicians make on a daily basis weighing heavily on what the literature has proven, statistical competency among practicing physicians has been largely unassessed.

A sound understanding of statistics and the interpretation of data are crucial in making decisions and predictions based on results presented in the literature. The purpose of this study was to assess the understanding of biostatistics in shoulder and elbow surgeons and current fellows. Specifically, we surveyed their ability to interpret data and identify statistical terminology. We also gathered their subjective attitudes toward statistics and confidence in understanding the various topics.

## 2. Methods

We conducted a cross-sectional survey that was administered to members of the American Shoulder and Elbow Surgeons (ASES) via email. All surveys were conducted online in an unmonitored environment. This study was approved by the Institutional Review Board for Human Studies at Orlando Health Medical Centre and the University of Central Florida College of Medicine (ORA#1640952). All data can be accessed via the deidentified Qualtrics data reporting service.

### 2.1. Survey Design

Participants were asked to complete all four sections of our survey. Sections included participant (1) demographics and education, (2) perception of statistics, (3) confidence in the ability to understand various statistical concepts, and (4) the biostatistics knowledge examination (BKE).

In the first section, we collected data regarding participant gender, age, fellowship subspeciality, years in practice, professional degrees held, training location, number of publications, and involvement with peer-review process and medical education (Table 1). In section two, we used a Likert scale (strongly disagree, disagree, neutral, agree, and strongly agree) to examine the opinions shared by participants as it relates to the value of statistics.  Section 3 evaluated the self-reported participant confidence in statistical interpretation. We used a five-point scale with zero symbolising no confidence and five symbolising full confidence.

Lastly, the fourth section included the BKE, which was developed by Windish et al.,and has been shown to offer good discriminative ability of statistics knowledge [13]. The examination tests commonly used methods and the understanding of terms encountered in statistics. The BKE consisted of 20 multiple-choice questions presented in a vignette-type fashion. No calculations were required. Windish adopted two questions from a Danish study with a similar focus [6]. Several were generated from the course material at John Hopkins Bloomberg School of Public Health [14], and the remainder tested concepts used in publications across six medicine journals with high impact factors (American Journal of Medicine, Annals of Internal Medicine, BMJ, JAMA, Lancet, and New England Journal of Medicine). The specific topics tested are shown in Table 2.

### 2.2. Data Collection and Analysis

A Qualtrics XM-based survey was administered by email to all members of the ASES and all responses were collected anonymously. Respondents were limited to practicing orthopaedic surgeons and orthopaedic surgery fellows.

An a priori statistical power analysis was performed and determined a sample size of 52 participants which was necessary to achieve a power of 0.8 (anticipated effect size = 0.7, probability level = 0.05). The survey was administered on January 5th, 2022, and remained open through February 2022. Participation was optional. Data were recorded and analysis was performed using *R* Core Team version 4.1.3 (*R* Foundation for Statistical Computing, Vienna, Austria). Participants received a report following survey completion, which provided a performance score for the BKE and identified correctly and incorrectly answered questions.

Only fully completed surveys were used in our analysis. Each question was analysed individually across the surveyed cohort. In addition, we assessed whether an association existed between various factors gathered from our other data points and BKE scores using bivariate and multivariate analyses. Variable selection for multivariate analyses was conducted using a forward stepwise selection to determine factors most strongly associated with correct BKE scores on univariate analyses [15]. Those with *p* values <0.1 were assessed in multivariate cohorts, grouped by demographic variables and confidence/attitude scores. Differences among participant characteristics and BKE performance were analysed using a student's *t*-test or a one-way analysis of variance (ANOVA). Participant characteristics were used as independent variables while the mean correct percentage on the BKE was evaluated as the dependent variable. Analyses were corrected for multiple comparisons to minimise the effect of random chance influencing the results. Variables with *p* values remaining ≤0.05 on all comparisons were deemed to remain significant without influence from random chance.

## 3. Results

### 3.1. Demographics

Of the 55 surgeons (4.5% response rate from distribution) who completed the survey, 92.7% were male and 80% received their medical training from within the United States. The highest proportion of participants was from 40 to 49 years (30.9%), followed by 30–39 years (27.3%). The majority of respondents reported to have been fellowship-trained in shoulder and elbow surgery (52.7%). In addition, most held a Doctor of Medicine (MD) designation (87.3%), and a quarter of these participants reported additional degrees. Over 70% reported publishing at least one manuscript per year, with 16% of all respondents publishing nine or more annually. Similarly, 52 of the participants (94.5%) had at one point been involved in the peer review process for various journals, and greater than 80% have had some degree of involvement with medical education (Table 1).

### 3.2. Biostatistics Knowledge Examination

Overall, 55.2% of questions on the BKE were answered correctly. Participants performed best when asked to interpret relative risk (96.4% correct) and in recognising a double-blind study (92.7% correct). The two areas where participants performed the weakest were when interpreting a 95% confidence interval and statistical significance (9.1%) and when asked to interpret Kaplan–Meier analysis results (12.7%) (Table 2). Cronbach's alpha of 0.78 demonstrated a good internal consistency of the BKE within the study population. This value indicates that participants who score well on most items are likely to continue to do so on the remaining items, as the test questions have high internal consistency.

### 3.3. Factors Associated with Statistical Knowledge

Younger participants scored significantly higher on the BKE compared to older examinees across all analysed age cohorts (*p* < 0.001). Additional statistically significant differences in BKE performances were identified when compared against time since medical school graduation and ABOS examination completion. Surgeons with more scholarly activity (>9 publications annually) were also found to have significantly higher scores on the BKE (*p*=0.009) (Table 1). Comparing scores among gender did not note a significant difference on the BKE. However, through logistic regression analysis, female gender, early career surgeons, and scholarly activity were predictors of higher BKE scores (*p* < 0.001, *p* < 0.001, and *p*=0.009, respectively). The proportion of explained variance for the models was large, with *R*^2^ = 0.60, indicating a high amount of score variance due to analysed factors.

### 3.4. Attitudes and Self-Reported Confidence

Participants overall shared a positive opinion on the value of statistics, with 98.2% agreeing that statistical competency is important and 80% favouring continued education in statistics. Over 90% of respondents reported that statistics help guide medical decision making in their practice. Attitudes toward statistics were not significantly associated with higher BKE scores (Table 3). Participants who reported high confidence in interpreting statistical results (*p*=0.009) and assessing the correct statistical test (*p*=0.017) demonstrated significantly higher BKE scores than those who did not. In contrast, participants who claimed high confidence in their ability to identify factors influencing the power of a study had significantly lower BKE scores (*p*=0.011) (Table 3).

## 4. Discussion

### 4.1. Background and Rationale

The present study employed a cross-sectional survey to assess the understanding and confidence members of the American Shoulder and Elbow Surgeons have in biostatistics. Reports have surfaced suggesting that care providers often misinterpret statistical methods and outcomes, which would question their ability to make sound evidence-based decisions [16]. It is thus timely and important to evaluate how fellowship-trained orthopaedic surgeons perceive their aptitudes for understanding biostatistics in the literature and how they perform when administered a BKE. Our study population largely consisted of academic orthopaedic shoulder and elbow surgeons, many of whom publish frequently (>70% reported publishing at least one manuscript per year and 16% publish 9 or more per year) or are a part of reviewing scientific research for publication, which includes critiquing of statistical analyses. It was concerning that this subset of surgeons scored 55.2% correctly on average using the BKE, as potentially flawed research may be distributed to other academic or community surgeons with errant conclusions often taken at face value. One could infer that if our study population scored only 55.2% correctly, nonacademic and community surgeons would likely score lower. When interpreting data to inform their practice, these surgeons may rely on studies' conclusions if they are unfamiliar with the statistical methodology. Furthermore, the recent surge in publication volume and complexity of statistical analyses only compound the aforementioned problems. When appropriate, the use of basic statistical methods and a thorough description of complex methods may improve reader understanding of study data. While continued medical education could benefit surgeons' understanding of biostatistics, it is unclear whether this would be sufficient with the evolving statistical complexity in studies or if the average orthopaedist would be interested. However, targeting journal reviewers with screening, testing, or other additional qualifications prior to commenting on a study's methodology may improve the clarity and correctness of statistical interpretation in orthopaedic literature.

### 4.2. Limitations

This study had several limitations, most of which were due to the survey format. Although many important concepts were covered in the BKE, the examination was limited to 20 questions and used topics commonly represented in medicine journals rather than orthopaedic surgery journals (e.g., Kaplan–Meier analysis is rarely utilised in orthopaedic studies and only 12.7% of our cohort answered this topic correctly). The limited volume of questions suggests that the current BKE may not accurately represent the true overall knowledge that our participants have in regard to biostatistics. The study population was limited to only ASES members, which included practicing surgeons and fellows in ASES-accredited programs; this limitation may have skewed results and may not be generalisable to the general orthopaedic surgery physician population. In addition, the present study found that women performed better than men on the BKE. This differs from results reported by Windish et al. [13], which found no difference. Further research is needed to understand if this is unique to the female members of the ASES or a generalisable finding. Volunteer bias may have also played a role for both the female and male participants, which may not be representative of the entire ASES membership.

The current questionnaire achieved only a 4.5% survey response rate which may introduce significant selection bias and reduce external validity. This selection bias may have also led to results that were not reflective of the majority of orthopaedic surgeons. The prior Windish et al. study using the BKE was administered in-person during residency noon conferences to assure attendance, with rates over 70% [13]. Due to the lack of incentive to complete the study questionnaire in our cohort and online administration, fewer responses were expected to be collected [1, 2]. Data errors resulting from omitted questions may skew final results; however, we did manage to achieve sufficient overall power with a sample size of 55. Lastly, this test was administered online for remote completion. This may have allowed participants to utilise outside resources to assist with answering questions and overestimated BKE scores.

### 4.3. Biostatistics Knowledge Examination

Naturally, some statistics concepts are more easily comprehended than others. Participants were better able to interpret relative risk and recognise a double-blind study than interpret Kaplan–Meier analysis results or a 95% confidence interval and the corresponding statistical significance. Only 27.3% of respondents could identify a Chi-squared test, which is essential for many orthopaedic studies, and 9.1% of respondents correctly interpreted 95% confidence intervals and statistical significance. This result is similar to that which Windish et al. reported [13], where 25.6% and 11.9% of surveyed internal medicine residents answered these questions correctly for Chi-squared and confidence intervals, respectively. In addition, the respondents in their study similarly performed best when asked to interpret relative risk and recognise a double-blind study. The above finding may come as a surprise considering that Chi-squared tests, confidence intervals, and claims for statistical significance are commonly encountered concepts in the literature. However, misinterpretation of confidence intervals has been previously reported [17]. The failure to accurately interpret these results for both our current analysis and by Windish et al. may be attributed to confusing verbiage used in the questions and answers themselves. The BKE used three questions to test participants' knowledge of these principles. Answers were non-numerical and required participants to select an answer from a list of distractors that were similarly worded, which required examinees to know the precise definition for the tested concepts. However, one could argue that testing the nuances and specifics of these concepts is critical to understanding and applying them to studies and that physician misinterpretation is a failure to understand these tested concepts. While these findings may raise concern, there is no evidence looking at the consequence that misinterpretation could have on a physician's ability to apply research findings safely and effectively into his or her own practice. Over 90% of our respondents reported using statistics to guide medical decision-making, though less than 10% tested correctly on interpreting confidence intervals and statistical significance. Furthermore, we reported that participants with higher confidence in their ability to interpret study power were associated with lower BKE scores. This false confidence could lead surgeons to errant conclusions and medical decision-making. Further research will be needed to determine if a novel question format could more accurately assess the ability to understand and interpret confidence intervals and claims of statistical significance.

### 4.4. Factors Associated with Statistical Knowledge

In 2020, applicants who successfully matched into a United States orthopaedic surgery residency had an average 14.3 publications, presentations, and posters, highlighting the early engagement orthopaedic surgeons have with research participation and interpretation [13]. However, Ngaage et al. found that in successfully matched orthopaedic surgery residents, the median number of publications was 1 and that 40% did not hold any publications, demonstrating a dichotomy between works reported and actually completed [18]. This finding may undermine the value of earlier exposure and participation in research of orthopaedic surgeons as there is a push for increased academic productivity at the cost of reduced project responsibility and a shift toward faster published articles.

The respondents in the current study were all fellowship-trained or currently in fellowship, which may offer additional opportunities for biostatistics education. Our study participants' large academic involvement may have resulted in higher scores than the average orthopaedic surgeon. Windish et al. [13] also discovered that BKE performance trended downward as individuals were further out from their formal medical education. This may suggest an opportunity for improvement in the continuing medical education structure. This has been addressed in recent years; medical education leaders have taken strides to incorporate biostatistics as a part of the formal curriculum for orthopaedic trainees [11, 19]. Further research will be needed to understand the individual effect of medical education on the understanding and interpretation of biostatistics for practicing surgeons.

### 4.5. Attitudes and Self-Reported Confidence

Our study demonstrated a consensus by 98% of respondents, which showed that statistical competency is important. However, BKE performance was poor overall with respondents averaging only 11 out of 20 questions. These findings indicate that future work should investigate methods to continue statistical education among orthopaedic surgeons.

## 5. Conclusions

This study assessed the biostatistical knowledge of fellowship-trained shoulder and elbow surgeons, many of whom frequently publish or are reviewing scientific literature for publication. This population scored an average of 55.2% correctly, raising concern that some of the most research-literate, academic shoulder, and elbow surgeons lack basic statistical understanding. This may yield implications that potentially flawed research is being distributed to other academic or community surgeons with subsequent errant conclusions. Future directions to improve research reliability and reader understanding include thorough descriptions of research methods and limitations of such used in studies, as well as utilisation of basic statistical methods when appropriate. While continuing medical education may also benefit orthopaedic surgeons, it is unclear if this would be sufficient or if the average orthopaedic surgeon would be interested. Targeting orthopaedic journal reviewers with screening or other additional qualifications prior to commenting on statistical methodology could improve the clarity of results published and distributed to orthopaedic surgeons at large.

Our study demonstrates that younger surgeons, female surgeons, and those with a greater number of publications per year scored higher on the BKE. Improved scores for younger respondents and those closer to their time in training may be due to familiarity with biostatistics and enhanced emphasis of statistical education in modern medical school, residency, and fellowship curricula. Further research is needed to understand the effect of gender, medical education, phase of career, speciality, and subspeciality on physicians' level of understanding of biostatistics.

Nearly all respondents felt that statistics are important; this current study highlights that further work is needed to educate surgeons on how to interpret biostatistics. Improving our ability to work with statistics will allow surgeon researchers to continue driving the field of orthopaedic surgery towards better and safer treatments.

## Figures and Tables

**Table 1:** Demographic data and correlation with biostatistical knowledge exam scores.

Characteristic	Response	No. (%)	Mean % correct	% (95% CI)	*p* value	Multivariate regression
Gender	Female	4 (7.3)	57.5	(42.3 to 72.5)	0.573	**<0.001**
	Male	51 (92.7)	52.0	(46.9 to 56.5)		

Age	30–39	15 (27.3)	63.5	(57.0 to 70.5)	**<0.001**	**<0.001**
	40–49	17 (30.9)	55.5	(47.6 to 63.5)		
	50–59	6 (10.9)	54.0	(43.9 to 64.5)		
	60–69	11 (20.0)	39.6	(30.4 to 48.8)		
	>70	6 (10.9)	35.0	(24.3 to 45.8)		

Fellowship completed	Shoulder and elbow	29 (52.7)	28.4	(19.0 to 37.7)	0.177	0.902
	Sports med	5 (9.1)	26.1	(5.0 to 47.3)		
	Multiple	8 (14.5)	29.6	(16.1 to 43.2)		
	Others	6 (10.9)	47.5	(38.5 to 56.5)		
	Hand and upper extremity	7 (12.7)	53.0	(32.1 to 73.5)		

Years since medical school	0–9	9 (16.4)	65.0	(56.5 to 73.5)	**0.001**	**0.001**
	10–19	20 (36.4)	57.0	(49.4 to 64.0)		
	20–29	8 (14.5)	54.5	(45.6 to 63.0)		
	30–39	12 (21.8)	42.1	(32.3 to 52.0)		
	>40	6 (10.9)	35.0	(24.3 to 45.8)		

Years since board exam	Not yet taken	9 (16.4)	55.0	(45.4 to 64.5)	**<0.001**	**<0.001**
	1–5	6 (10.9)	68.5	(61.5 to 75.5)		
	6–10	8 (14.5)	66.0	(54.5 to 78.0)		
	11–15	11 (20.0)	52.5	(43.4 to 61.0)		
	16–20	2 (3.6)	60.0	(40.4 to 79.5)		
	>20	19 (34.5)	39.0	(32.6 to 45.3)		

Degrees	DO	1 (1.8)	27.5	(26.4 to 81.5)	0.422	0.198
	MD	36 (65.5)	34.7	(27.2 to 42.3)		
	MD + others	12 (21.8)	40.3	(25.4 to 55.0)		
	Others	6 (10.9)	53.5	(45.1 to 61.5)		

Training outside of USA	Yes	11 (20.0)	36.8	(22.7 to 51.0)	0.95	0.676
	No	44 (80.0)	37.4	(30.4 to 48.8)		

Average papers published per year	Less than 1	16 (29.1)	48.1	(33.3 to 52.0)	**0.009**	**0.009**
	1-2	11 (20.0)	42.8	(49.4 to 67.5)		
	3–5	15 (27.3)	58.5	(25.1 to 57.5)		
	6–8	4 (7.3)	41.3	(56.5 to 74.5)		
	9+	9 (16.4)	65.5	(40.6 to 55.5)		

Involvement in peer review process	Yes	52 (94.5)	52.5	(47.6 to 57.5)	0.583	0.583
	No	3 (5.5)	46.7	(43.4 to 50.0)		

Involvement of medical education	Yes	45 (81.8)	53.0	(47.8 to 58.0)	0.471	0.471
	No	10 (18.2)	48.5	(37.6 to 59.5)		

Receiving NIH funding	Yes	7 (12.7)	48.6	(33.4 to 64.0)	0.181	0.181
	No	41 (74.5)	51.0	(45.5 to 56.5)		
	Other funding	7 (12.7)	63.5	(55.5 to 72.0)		

Bold values represent statistically significant results.

**Table 2:** Percentage of correct answers for biostatistical knowledge examination.

Question	Objective	No. correct %	CI %
1a	Identify continuous variable	76.4	(63.5 to 85.8)
1b	Identify ordinal variable	58.2	(45.0 to 70.3)
1c	Identify nominal variable	45.5	(33.0 to 58.5)
2	Recognise a case-control study	54.6	(41.5 to 67.0)
3	Recognise purpose of double-blind studies	92.7	(82.3 to 97.6)
4a	Identify ANOVA	60	(46.8 to 71.9)
4b	Identify chi squared test	27.3	(17.2 to 40.3)
4c	Identify *t* test	76.4	(63.5 to 85.8)
5	Recognise definition of bias	47.3	(34.7 to 60.2)
6	Interpret the meaning of *p* value >0.05	54.6	(41.5 to 67.0)
7	Identify cox proportional hazard regression	16.4	(8.6 to 28.5)
8	Interpret standard deviation	69.1	(55.9 to 79.8)
9	Interpret 95% CI and statistical significance	9.1	(3.5 to 20.0)
10	Recognise power, sample size, and significance-level relationship	52.7	(39.8 to 65.3)
11	Determine which test has more specificity	63.6	(50.4 to 75.1)
12	Interpret an unadjusted odds ratio	72.7	(59.7 to 82.8)
13	Interpret odds ratio in multivariate regression analysis	43.6	(31.4 to 56.7)
14	Interpret relative risk	96.4	(87.0 to 99.7)
15	Determine strength of evidence for risk factors	74.6	(61.6 to 84.3)
16	Interpret Kaplan–Meier analysis results	12.7	(6.0 to 24.3)

**Table 3:** Attitudes and confidence of statistical competency.

Characteristic	Response	N (%)	Mean % correct	% (95% CI)	*p* value	Multivariate regression
Would like to learn more about statistics	Strongly disagree	1 (1.8)	50.0	(38.6 to 61.5)	0.29	0.29
	Neither agree or disagree	10 (18.2)	48.6	(40.8 to 56.5)		
	Somewhat agree	18 (32.7)	54.5	(47.7 to 61.0)		
	Strongly agree	26 (47.3)	80.0			

Thinks statistical competency is important	Neither agree or disagree	1 (1.8)	45.0		0.606	0.606
	Somewhat agree	26 (47.3)	50.0	(42.4 to 57.5)		
	Strongly agree	28 (50.9)	54.5	(48.7 to 60.0)		

I often use statistical information in forming opinions	Neither agree or disagree	4 (7.3)	40.0	(29.4 to 50.5)	0.384	**0.003**
	Somewhat agree	30 (54.5)	52.5	(46.3 to 59.0)		
	Strongly agree	21 (38.2)	54.0	(46.1 to 62.0)		

Increased emphasis on statistical analysis should be examined in the ABOS	Strongly disagree	10 (18.2)	44.5	(34.9 to 54.0)	0.133	0.13
	Neither agree or disagree	19 (34.5)	57.0	(49.6 to 62.0)		
	Somewhat agree	17 (30.9)	56.0	(47.6 to 66.5)		
	Strongly agree	9 (16.4)	57.0	(45.6 to 66.5)		

Confidence interpreting probability values	Strongly disagree	4 (7.3)	47.2	(26.8 to 67.5)	0.054	0.055
	Neither agree or disagree	7 (12.7)	51.5	(45.7 to 57.0)		
	Somewhat agree	30 (54.5)	36.3	(25.4 to 47.1)		
	Strongly agree	14 (25.5)	61.0	(54.0 to 68.0)		

Confidence in interpreting statistical results	Strongly disagree	3 (5.5)	42.0	(21.8 to 62.0)	**0.008**	**0.009**
	Neither agree or disagree	5 (9.1)	50.5	(45.1 to 56.0)		
	Somewhat agree	35 (63.6)	65.5	(24.9 to 48.5)		
	Strongly agree	12 (21.8)	36.7	(57.5 to 73.0)		

Confidence in identifying factors that influence the power of a study	Strongly disagree	9 (16.4)	56.5	(38.5 to 75.0)	**0.011**	**0.011**
	Neither agree or disagree	26 (47.3)	52.0	(45.4 to 58.5)		
	Somewhat agree	3 (5.5)	36.7	(28.7 to 44.7)		
	Strongly agree	17 (30.9)	60.0	(52.0 to 68.0)		

Confidence in assessing the correct statistical test	Strongly disagree	5 (9.1)	44.3	(30.2 to 58.5)	**0.007**	**0.017**
	Somewhat disagree	22 (40.0)	62.0	(55.0 to 69.0)		
	Neither agree or disagree	7 (12.7)	45.0	(37.8 to 52.0)		
	Somewhat agree	17 (30.9)	66.0	(61.5 to 71.0)		
	Strongly agree	4 (7.3)	50.0	(36.9 to 63.0)		

Bold values represent statistically significant results.

## Data Availability

The data used in this study are available on request to AC and BS.
